# Non-contrast T1rho CMR for hypertrophic cardiomyopathy

**DOI:** 10.1186/1532-429X-17-S1-P273

**Published:** 2015-02-03

**Authors:** Chunhua Wang, Jie Zheng, Jiayu Sun, Yuqing Wang, Rui Xia, Qian Yin, Wei Chen, Jichun Liao, Bing Zhang, Fabao Gao

**Affiliations:** Department of Radiology, West China Hospital, Sichuan University, Chengdu, China; Mallinckrodt Institute of Radiology, Washington University School of Medicine, St. Louis, MO USA; CAS Key Laboratory for Biomedical Effects of Nanomaterials and Nanosafety, National Center for Nanoscience and Technology of China, Beijing, China; Department of Radiology, The First Affiliated Hospital of Chongqing Medical University, Chongqing, China

## Background

Hypertrophic cardiomyopathy (HCM) is a genetic disease characterized by heterogeneous clinical symptoms and able to be visualized by cardiac magnetic resonance (CMR). Late gadolinium enhancement (LGE) represents myocardial fibrosis. There is still a challenge for HCM patients with contrast agent contraindication. T1rho CMR has been reported to determine fibrosis in myocardial infarction. To our best knowledge, no T1rho studies in HCM patients reported. We aimed to explore whether T1rho CMR detects fibrosis in HCM patients.

## Methods

Twelve HCM patients underwent T1rho imaging using an ECG-gated T1rho prepared gradient echo(GRE) sequence before LGE imaging. Three short-axis views of T1rho and LGE sequences were obtained from base to apex. T1rho imaging parameters were: TR 3.2 ms, TE 1.52 ms, flip angle 15°. FOV 172 mm × 232 mm, voxel size 1.8 mm × 1.8 mm × 8.0 mm, time of spin locking = 10, 30, 50 ms, and spin locking frequency = 340.6 Hz. Images were analyzed by a custom-made software written in Matlab 7.1. for T1rho relaxation time map. T1rho relaxation times in LGE area and remote area were measured by ImageJ.

## Results

**Figure 1:**
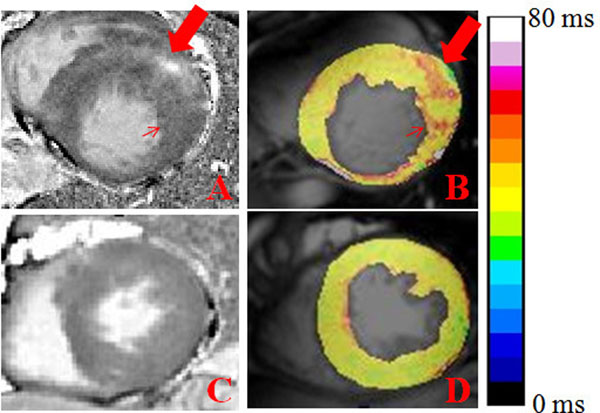
T1rho relaxation time and LGE images.(A-D) The LGE area (thick arrow in A) showed higher T1rho value than area without LGE (B and C). However, hyper-T1rho value (thin arrow in B) also presented in some area without LGE(thin arrow in A). LGE, late gadolinium enhancement.

LGE was present in 8 patients. T1rho relaxation times of scarring and remote myocardium were summarized in Table 1. The mean T1rho values of LGE and remote areas were 60.2 ms and 42.8 ms respectively. LGE area has the tendency to show higher T1rho than remote area. Because of small sample, no statistical analysis was taken. Furthermore, hyper-T1rho value also located in the area without LGE (Fig. 1).

## Conclusions

LGE area showed hyper-T1rho values, suggesting T1rho has a potential to detect myocardial fibrosis in HCM patients. Hyper-T1rho values in area without LGE might be explained by diffuse fibrosis. In conclusion, non-contrast T1rho might visualize myocardial fibrosis includes diffuse fibrosis regardless contrast agent contraindication.

## Funding

This work was supported by the National Natural Science Foundation of China [grant number81130027; 81071204][http://www.nsfc.gov.cn/Portal0/default166.htm], and the National Basic Research Program of China [973 Program, grant number 2011CB935800] [http://www.973.gov.cn/English/Index.aspx].

**Table 1:** T1rho relaxation times

Patient	1	2	3	4	5	6	7	8	9	10	11	12
T1rho-value of LGE area (ms)	57.4	59	60.9	60.7	63.1	55.8	61.8	62.7	N	N	N	N
T1rho-value of remote area (ms)	42.4	40.3	43.9	44.1	41.9	42.1	41.9	42.6	42.8	42.3	44.1	45.7

LGE, late gadolinium enhancement; N, none.

